# Exploring potential associations between low-dose cardiac catheterization radiation and blood microRNA changes in children with congenital heart disease

**DOI:** 10.1093/carcin/bgag032

**Published:** 2026-05-25

**Authors:** Jonica Campolo, Giuseppe Annoni, Andrea Borghini, Alessio Peretti, Marco Papa, Lamia Ait-Ali, Marie-Odile Bernier, Estelle Rage, Jérémie Dabin, Isabelle Thierry-Chef, Siamak Haghdoost, Eugenio Picano, Maria Grazia Andreassi

**Affiliations:** CNR Institute of Clinical Physiology, Milan 20162, Italy; Pediatric Cardiology, ASST Grande Ospedale Metropolitano Niguarda, Milan 20162, Italy; Pediatric Cardiology, Regina Margherita Children's Hospital, Turin 10126, Italy; CNR Institute of Clinical Physiology, Via Moruzzi 1, Pisa 56124, Italy; Pediatric Cardiology, ASST Grande Ospedale Metropolitano Niguarda, Milan 20162, Italy; Pediatric Cardiology, ASST Grande Ospedale Metropolitano Niguarda, Milan 20162, Italy; CNR Institute of Clinical Physiology, Via Moruzzi 1, Pisa 56124, Italy; Laboratory of Epidemiology, Autorité de Sûreté Nucléaire et de Radioprotection, Fontenay-Aux-Roses 92262, France; Laboratory of Epidemiology, Autorité de Sûreté Nucléaire et de Radioprotection, Fontenay-Aux-Roses 92262, France; Belgian Nuclear Research Centre, SCK CEN, Mol 2400, Belgium; Barcelona Institute for Global Health—ISGlobal, Barcelona 08036, Spain; University of Stockholm, Stockholm SE-106 91, Sweden; ABTE/ToxEMAC Laboratory, University of Caen Normandy, Caen 14032, France; Clinical Center of Serbia and School of Medicine University of Belgrade, Belgrade 11000, Serbia; CNR Institute of Clinical Physiology, Via Moruzzi 1, Pisa 56124, Italy

**Keywords:** congenital heart disease, cancer risk, ionizing radiation, microRNA

## Abstract

The risk of cancer from medical exposure to ionizing radiation (IR) in pediatric patients—particularly those with congenital heart disease (CHD)—is a concern, given the need for repeated X-ray-guided cardiac catheterization (CC) procedures throughout their lives. To better understand the effects of low-dose IR, this study—part of the European HARMONIC project—investigated changes in blood miRNA expression following CC in CHD patients. Small RNA sequencing was performed on a discovery cohort of 10 CHD patients at three time points: before CC (T_0_), immediately after (T_1_), and 1 year later (T_2_). Differentially expressed miRNAs were validated in an independent group of 15 patients using quantitative real-time polymerase chain reaction (qRT-PCR). Kyoto Encyclopedia of Genes and Genomes pathway annotation analysis was carried out to determine the biological pathways involving target genes regulated by the dysregulated miRNAs. At the discovery stage, 74 miRNAs were significantly differentially expressed between the T_1_ and T_0_ groups, 69 between the T_2_ and T_0_ groups, and 41 between the T_2_ and T_1_ groups (*q*-value < 0.05 and |log2(fold change)|>1). Sixteen miRNAs (9 upregulated and 7 downregulated) were found to be dysregulated in both T_1_ and T_2_ compared to T_0_. Additionally, seven known miRNAs were uniquely dysregulated at T_2_. qRT-PCR analysis of six selected miRNAs partially validated the sequencing results, confirming consistent expression patterns for four miRNAs across cohorts. Pathway enrichment analysis indicated that predicted miRNA target genes were overrepresented in several pathways, including those annotated as cancer-related (enrichment *P* < .0001). These findings suggest potential associations with radiation exposure, but their biological significance and health implications remain uncertain and require further investigation.

## Graphical Abstract

**Graphical Abstract:**
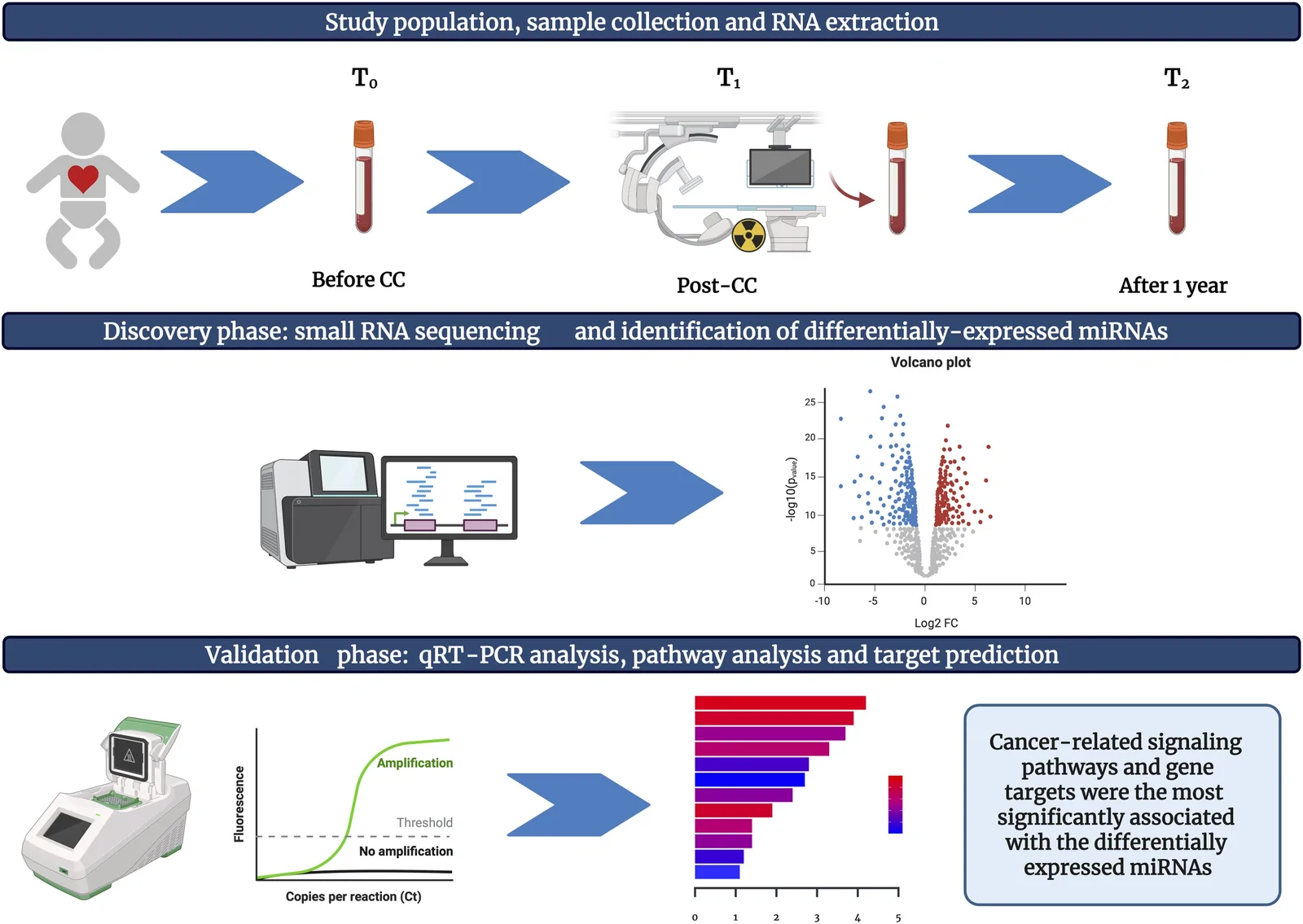
Low-dose ionizing radiation from cardiac catheterization alters blood microRNA profiles linked to cancer pathways in children with congenital heart disease

## 1. Introduction

The risk of cancer due to medical exposure to ionizing radiation (IR) in pediatric patients is a critical concern due to the higher sensitivity of children’s tissues and their longer life expectancy, which allows more time for potential radiation-induced cancers to develop [1, 2].

This risk may be higher in patients with congenital heart disease (CHD) due to repeated X-ray-guided procedures over their lifetime, which increase cumulative radiation exposure [1, 2].

Additionally, certain genetic conditions associated with CHD (e.g. Down syndrome) confer an intrinsically increased risk of malignancy. Repeated exposure to IR from diagnostic imaging or therapeutic procedures may further amplify this risk beyond the underlying genetic predisposition [2].

Recent epidemiological studies have demonstrated an association between childhood exposure to computed tomography (CT) scans and an increased risk of cancer, with particularly elevated risks observed for hematological malignancies and brain tumors [3, 4]. A large population-based study also reported a link between low-dose IR from cardiac procedures and increased cancer incidence in adults with CHD, especially among those undergoing a higher number of diagnostic and therapeutic interventions [5]. However, studies focusing on pediatric CHD patients have not found a significant increase in cancer risk related to radiation dose [6, 7]. Nonetheless, the statistical power of these studies was limited due to small sample sizes and the relatively low expected risk. In order to increase the statistical power of epidemiological analyses, larger studies are needed to more accurately assess the risks associated with pediatric exposure in patients with CHD. Accordingly, the HARMONIC (Health effects of Cardiac Fluoroscopy and Modern Radiotherapy in Pediatrics) project aims at complementing existing studies on CT scan-related radiation exposure [3, 4] by investigating the health effects and underlying biological mechanisms of medical ionizing exposure in childhood and adolescence [8].

Through its cardiology component, the project brings together large national cohorts from across Europe involving patients who underwent cardiac catheterization (CC) at a pediatric age to investigate cancer risk following exposure to IR with a level of precision unattainable in individual national studies [6–8].

In addition, comprehensive biological research will integrate the epidemiological findings by providing mechanistic insights and complementary data [9].

To more accurately assess the cancer risks associated with low-dose exposures, it is essential to understand the mechanisms by which low-dose radiation induces cellular and molecular damage, as well as how the body responds and repairs this damage [9, 10].

Exposure to IR, even at low doses, can lead to various cellular responses such as DNA damage, oxidative stress, apoptosis, and alterations in cell cycle regulation [9]. MicroRNAs (miRNAs or miR) play a crucial role in governing the regulation of both transcriptional and posttranscriptional gene expression associated with DNA repair, apoptosis, oncogenes, and tumor suppressor genes [11]. Understanding miRNA expression changes in response to radiation can provide insights into the mechanisms of radiation-induced carcinogenesis and help in risk assessment and early detection [12].

The main objective of this study was to investigate the dynamic expression profile of miRNAs after CC procedures in blood samples of CHD patients.

We employed small RNA sequencing for an unbiased analysis to identify different miRNA signatures in response to radiation exposure. This approach also allowed us to assess their pathophysiological relevance, shedding light on the pathways and biological processes associated with the target genes.

## 2. Materials and methods

### 2.1. Patient population

As part of the HARMONIC Project, the current study included patients who underwent CC procedures and were recruited between January 2015 and October 2023 at Niguarda Hospital in Milan, Italy [13]. Patients were considered eligible for inclusion if they underwent at least one CC procedure at an age < 22 years [13]. Exclusion criteria included a history of cancer diagnosis before the first CC procedure. Informed consent was obtained from all patients or their parents, and the study was approved by the local ethics committee. Dose-area product (Gy cm^2^) and fluoroscopic time (min) were directly recorded for each patient during the procedure by the angiography system.

This study protocol consisted of two parts. In the initial screening phase, we performed small RNA sequencing on a discovery group of 10 patients with CHD to identify candidate dysregulated miRNAs after the CC procedure. In the subsequent validation phase, we further assessed selected differentially expressed (DE) miRNAs using quantitative real-time polymerase chain reaction (qRT-PCR) in a separate group of 15 CHD patients. Nine patients in the discovery group and three in the validation group underwent CC for diagnostic purposes.

A total of 13 patients were referred for therapeutic CC procedures: 9 for transcatheter closure of an atrial septal defect (*n* = 6) or of a patent ductus arteriosus (*n* = 3); 2 for stent implantation to treat coarctation of the aorta; and 2 for intervention on pulmonary stenosis, either by balloon angioplasty (*n* = 1) or pulmonary valvuloplasty (*n* = 1).

There were no significant complications during or after CC in either group, and most patients were discharged on the second day of the procedure. The baseline and procedural characteristics of our patients are shown in Table 1.

**Table 1:** Demographic and procedural characteristics of the study population.

Discovery group		Validation group
Number of patients	10	15
Age, years (mean ± SD)	11.2 ± 4.9	11.2 ± 9.4
Gender, *n*		
Male	7	8
Female	3	7
Procedure type		
Diagnostic CC, *n*	3	9
Therapeutic CC, *n*	7	6
DAP, Gy cm^2^ (median, IQR)	0.94 (0.1, 22.3)	3.3 (0.2, 49.2)
FT, min (median, IQR)	4.9 (1–18)	12.4 (0.5–70)

DAP, dose area product; FT, fluoroscopy time; IQR, interquartile range.

### 2.2. Blood sample collection and RNA extraction

Blood samples were collected at three time points: immediately before (T_0_), immediately after the procedure (T_1_), and ∼ 1 year after the procedure (T_2_).

The study design and schematic workflow of the overall analysis and experimental protocol are shown in Supplementary Fig. S1.

Total RNA from 300 μl whole blood was extracted using the RiboPure™ RNA Purification Kit (Thermo Scientific, Waltham, MA, USA) according to the manufacturer’s recommendations. The RNA concentration of each sample was determined with a Nanodrop Lite spectrophotometer (Thermo Scientific, Waltham, MA, USA), and the quality of RNA was assessed using the RNA 6000 Nano Kit with Bioanalyzer 2100 (Agilent Technologies, Santa Clara, CA, USA). Only samples with an absorbance ratio at 260/280 of > 1.7 and an RNA Integrity Number of ≥ 7.0 were analyzed.

### 2.3. Library preparation and small RNA sequencing

Small RNA sequencing was performed in Novogene Biotech (London, UK) as outsourcing. The methods, including library preparation and data analysis, are described as follows, according to the reports produced by Novogene Biotech. Briefly, 3’ and 5’ adaptors were ligated to the 3’ and 5’ ends of the small RNA, respectively. Then, the first strand cDNA was synthesized after hybridization with the reverse transcription primer. The double-stranded cDNA library was generated through PCR enrichment. The library was checked with Qubit and real-time PCR for quantification and a bioanalyzer for size distribution detection. Sequencing was performed on an Illumina HiSeq 2500/2000 platform, and 50 bp single-end reads were generated.

### 2.4. Data analysis

Raw data (raw reads) of fastq format were first processed through custom Perl and Python scripts. The small RNA tags were mapped to the reference sequence using Bowtie, an ultrafast and memory-efficient program for aligning short DNA sequence reads to large genomes [14].

MiRNA expression levels were estimated by transcript per million through the following normalized formula: Normalized expression = (Mapped reads/Total reads) × 10^6^. The edgeR software was used to screen the DE miRNAs [15]. Significantly dysregulated miRNAs between the two groups. were identified at *P*-values < .05 and log2 (fold change) > 1. The miRNAs with log2 (fold change) > 1 were labeled as upregulated miRNAs (Up), while miRNAs with log2 (fold change) < 1 were labeled as downregulated miRNAs (Down). Using the Benjamini–Hochberg multiple testing correction [16], miRNAs were considered statistically significant at *q*-value ≤ .05 (false discovery rate-corrected *P*-value).

### 2.5. qRT-PCR validation of differentially expressed miRNAs

Isolated total RNA was reverse transcribed into cDNA by using TaqMan miRNA Reverse Transcription Kit (Thermo Fisher Scientific, USA). Real-time PCR quantification of miRNAs was performed using sequence-specific TaqMan MicroRNA Assays and TaqMan Universal PCR Master Mix, no AmpErase UNG (Thermo Fisher Scientific, USA) following manufacturer’s instructions. qRT-PCR reactions were run in triplicate for each sample using a 384-well plate CFX RT-PCR System (Bio-Rad, Milan, Italy). The expression levels of miRNAs were normalized to U6 snRNA (ID:001973) by using the ΔΔ*Ct* method. Subsequently, RT-qPCR was performed using sequence-specific TaqMan MicroRNA Assays (Thermo Fisher Scientific, USA) and TaqMan Universal PCR Master Mix (no AmpErase® UNG) following the manufacturer’s instructions. The expression levels of miRNAs were normalized to U6 small nuclear RNA (ID:001973) by using the 2^−**ΔΔ*Ct***^ method.

### 2.6. Target prediction and pathway analysis

The DIANA tool miRPath v.4 was used to perform the pathway enrichment analysis by the simultaneous analysis of the combinatorial effect of DE miRNAs, based on the Kyoto Encyclopedia of Genes and Genomes (KEGG) database and Gene Ontology (GO) annotations [17]. The target genes of miRNA were identified using TargetScan v8.0 databases. The settings of the software were enrichment *P*-value threshold = .001, and the FDR correction filters were ticked.

### 2.7. Statistical methods

Continuous variables were compared using unpaired *t*-tests for parametric data and the Mann–Whitney test for nonparametric data. Categorical data, expressed as frequencies and percentages, were compared using a Fisher’s exact test. All tests were two-sided, and a *P*-value < .05 was considered statistically significant.

## 3. Results

### 3.1. MiRNA sequencing and quality check of the raw data

All 30 blood samples, collected at three different time points from the first 10 patients, were initially analyzed as the discovery group. Following library preparation and sequencing, the raw data were filtered and checked for errors. Regarding RNA data generation, we obtained between 8 and 18 million reads (Supplementary Table S1). In terms of quality metrics, the average values for Q20 (1 error per 100 bp) and Q30 (1 error per 1000 bp) were 99% and 96.9%, respectively, demonstrating high accuracy in base calling and sequencing. After trimming the Illumina adapters from the miRNA reads, most of the reads were approximately 22 bp in length (Supplementary Fig. S2).

### 3.2. Identification of differentially expressed miRNAs

**Figure 1:**
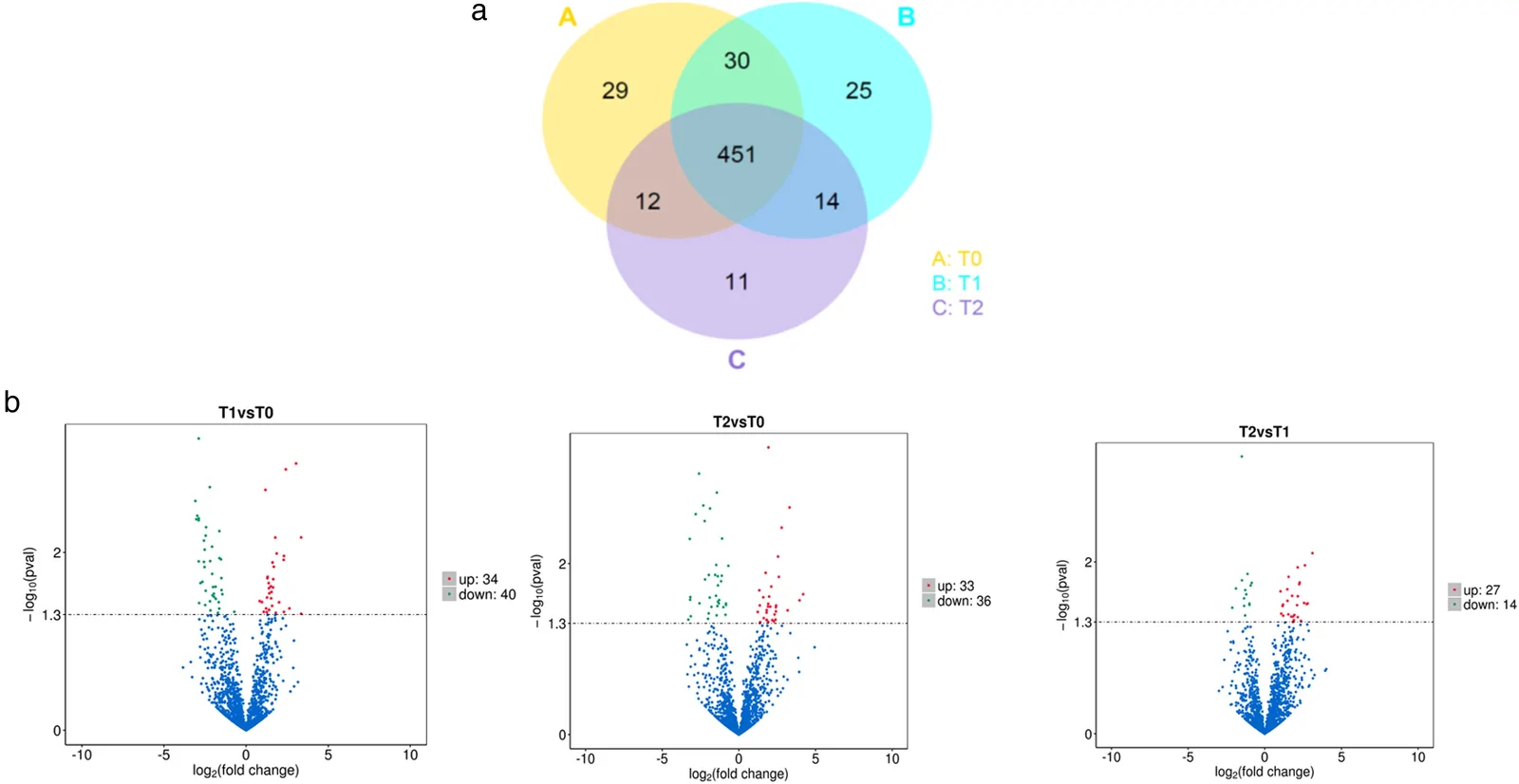
(a) Venn diagram of miRNAs in expression profiling datasets, (b) Volcano plot analysis of dysregulated miRNAs in samples according to different times. MiRNAs with significant differences (*P* value < .05) are represented by red (upregulation) and green dots (downregulation), and miRNAs without significant differences are represented by blue dots.

The total number of small RNAs ranged from 6.6 to 15.6 million, among which 1582 sRNAs aligned to known mature miRNA sequences in at least one patient. The Venn diagram (Fig. 1) illustrates that 451 miRNAs were commonly identified across all groups, while also displaying the miRNAs uniquely expressed within each group, and those shared between two groups. Among these, we identified 74 DE miRNAs between the T_1_ and T_0_ groups (34 upregulated, 40 downregulated), 69 DE miRNAs between the T_2_ and T_0_ groups (33 upregulated, 36 downregulated), and 41 DE miRNAs between the T_2_ and T_1_ groups (27 upregulated, 14 downregulated). We further searched for miRNAs that were dysregulated in both T_1_ and T_2_ compared with the pre-procedure baseline (T_0_). Sixteen known miRNAs were found to be significantly dysregulated at both time points following the CC procedures (Table 2). Additionally, seven known miRNAs were significantly dysregulated only 1 year after the CC procedure (T_2_), compared with both T_1_ and T_0_ (Table 3).

**Table 2:** Differentially miRNAs expressed in both T_1_ and T_2_ compared to T_0._

hsa-miRNA	T_1_ vs. T_0_			T_2_ vs. T_0_		
	Regulation	Log2 fold change	*q* value	Regulation	Log2 fold change	*q* value
hsa-miR-125a-3p	Up	3.04	0.002	Up	1.81	0.043
hsa-miR-5584-5p	Up	2.41	0.008	Up	1.82	0.043
hsa-miR-6780a-3p	Down	−2.88	0.020	Down	−3.20	0.016
hsa-miR-144-3p	Down	−2.07	0.032	Down	−2.23	0.012
hsa-miR-4738-3p	Up	1.86	0.032	Up	1.75	0.023
hsa-miR-760	Up	2.30	0.032	Up	3.30	0.011
hsa-miR-374a-3p	Down	−1.62	0.032	Down	−1.40	0.043
hsa-miR-32-5p	Down	−1.53	0.032	Down	−1.36	0.035
hsa-miR-4669	Up	2.29	0.032	Up	2.56	0.021
hsa-miR-6818-3p	Down	−2.06	0.032	Down	−1.96	0.043
hsa-miR-6815-5p	Up	1.39	0.035	Up	1.93	0.006
hsa-miR-619-5p	Up	1.55	0.035	Up	1.63	0.043
hsa-miR-374a-5p	Down	−1.44	0.035	Down	−1.40	0.011
hsa-miR-150-3p	Up	1.27	0.035	Up	1.39	0.050
hsa-miR-1278	Down	−1.84	0.035	Down	−1.89	0.035
hsa-miR-4784	Up	2.00	0.040	Up	2.39	0.043

**Table 3:** Differentially expressed miRNAs in T_2_ compared to both T_1_ and T_0._

hsa-miRNA	T_2_ vs. T_0_			T_2_ vs. T_1_		
	Regulation	Log2 fold change	*q* value	Regulation	Log2 fold change	*q* value
hsa-let-7f-5p	Down	−1.27	0.028	Down	−1.48	0.018
hsa-miR-103b	Down	−1.00	0.030	Down	−0.84	0.030
hsa-miR-146b-5p	Down	−0.92	0.028	Down	−1.10	0.018
hsa-miR-155-5p	Down	−1.50	0.007	Down	−1.11	0.023
hsa-miR-20a-5p	Down	−1.12	0.028	Down	−1.44	0.007
hsa-miR-4742-5p	Down	−1.88	0.028	Down	−2.20	0.023
hsa-miR-6868-3p	Down	−2.12	0.030	Down	−2.81	0.011

### 3.3. qRT-PCR validation of differentially expressed miRNAs

To confirm the accuracy of the miRNA expression identified by next-generation small RNA sequencing, we further validated a panel of miRNAs using qRT-PCR in an independent validation group of 15 additional patients. We selected five miRNAs that were dysregulated in both T_1_ and T_2_ compared to T_0_ (hsa-miR-32-5p, hsa-miR-125a-3p, hsa-miR-144-3p, hsa-miR-374a-3p, and hsa-miR-760), along with one additional miRNA (hsa-let-7f-5p) previously identified as highly dysregulated in T_2_ relative to both T_1_ and T_0_.

**Figure 2:**
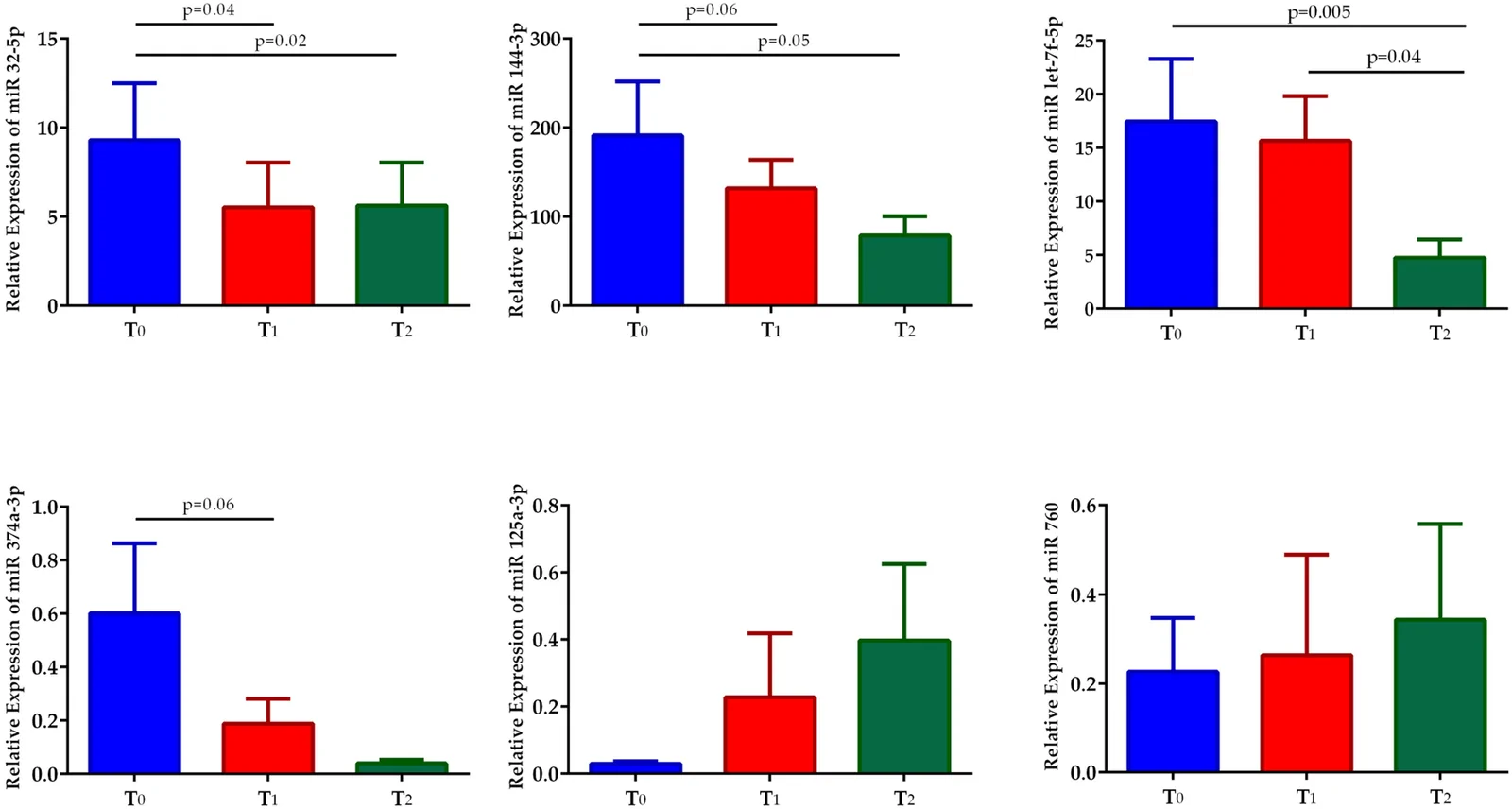
Validation of significantly differentially expressed miRNAs by qPCR.

The selection was based on their extensive research in the context of human cancers and radiation response. Overall, the expression of all these miRNAs showed consistent trends in the direction of regulation and expression pattern with the sRNA sequencing results at each time point after CC (Fig. 2). In particular, our results indicated that hsa-miR-32-5p was significantly downregulated in both T_1_ (*P* = .04) and T_2_ (*P* = .02) compared to T_0_. Additionally, hsa-let-7f-5p expression was markedly decreased at T_2_ compared to both T_1_ (*P* = .03) and T_0_ (*P* = .005).

Although in the same direction as miRNA sequencing, 2 miRNAs (hsa-miR-760, hsa-miR-125a-3p) did not reach significance because they were below the detection limit in some samples. In a correlation analysis, no significant correlations were found between miRNA expression and demographic characteristics or dosimetric parameters.

### 3.4. Functional analysis of most significantly differentially expressed miRNAs

To investigate the pathophysiological relevance of the DE miRNAs, a functional enrichment analysis of overrepresented pathways for the target gene sets and GO terms was performed independently for the two sets of miRNAs identified (16 DE miRNAs at both time points after CC procedures and 6 DE miRNAs 1 year after the procedure compared to baseline and immediately after the procedure).

**Figure 3:**
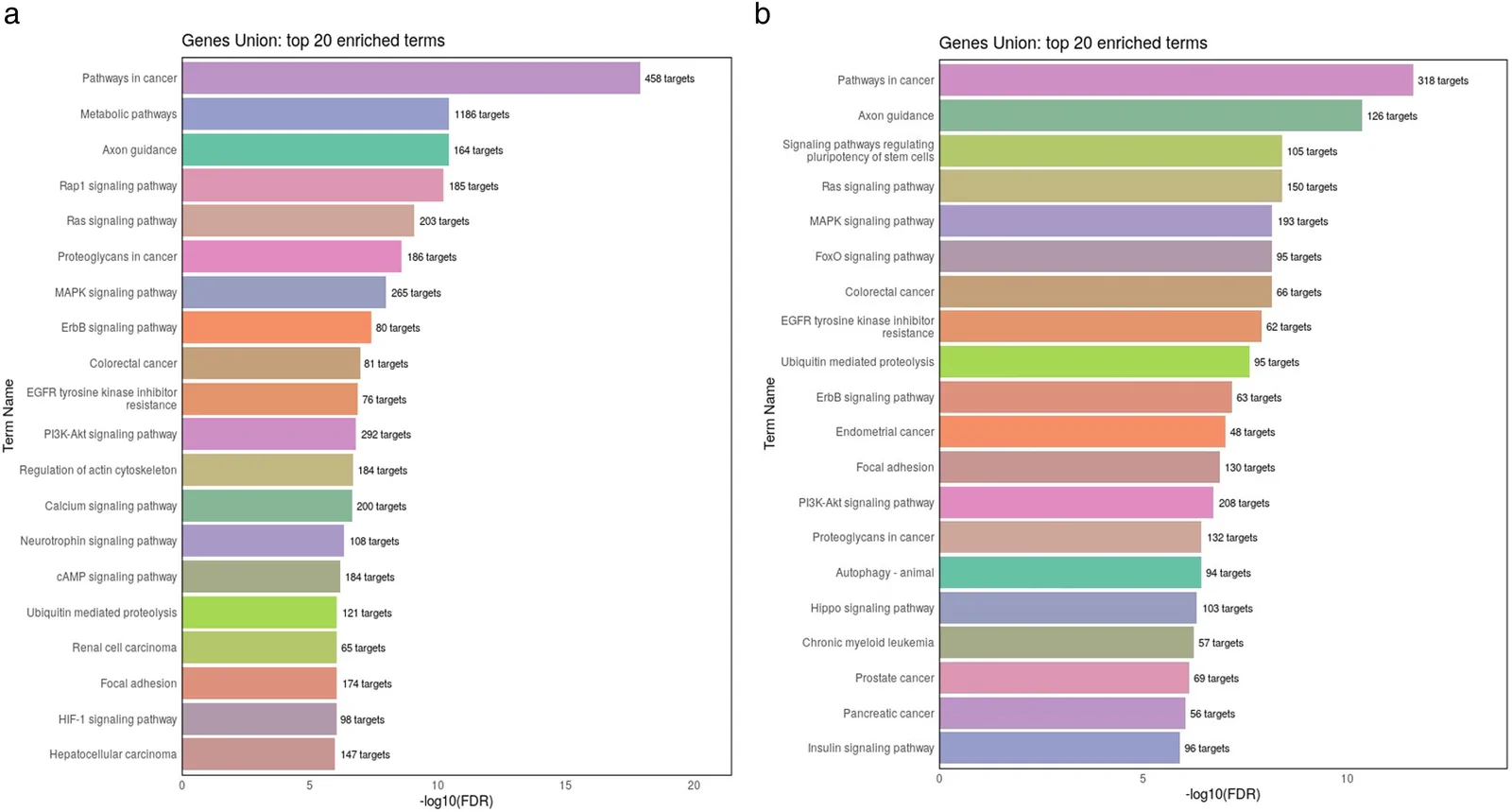
Top 20 significantly analyzed pathways for the predicted target genes related to the differentially expressed miRNAs: (a) 16 DE miRNAs at both times after CC procedures; (b) 6 DE miRNAs after 1 year from the procedure compared to baseline and immediately after the procedure.

The analysis revealed significant enrichment in 152 and 121 KEGG pathways for the target genes of 16 DE miRNAs and 6 DE miRNAs, respectively, with the top 20 statistically significant pathways presented in Fig. 3.

According to the KEGG analysis, the most significantly enriched pathway was the cancer pathways for both 16 DE miRNAs and 6 DE miRNAs (*P* = 3.6 × 10^−21^ and *P* = 7.1 × 10^−15^, respectively).

**Figure 4:**
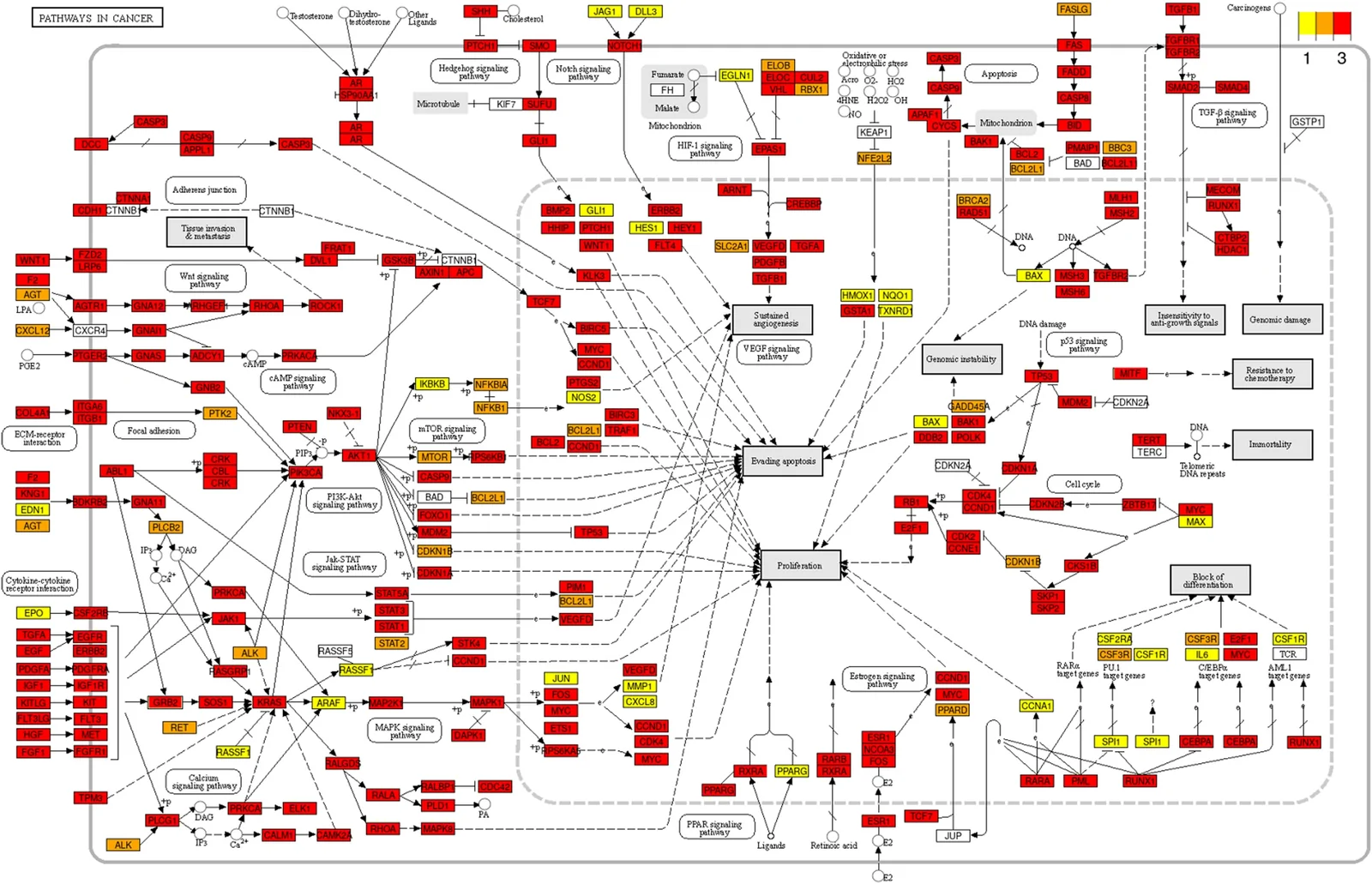
KEGG cancer-related pathways showing predicted target genes of 16 differentially expressed miRNAs. Cancer-related KEGG pathways with predicted target genes from 16 DE miRNAs. Genes targeted are color-coded: yellow for targets of one miRNA, orange for two, and red for three. Solid arrows indicate activation, dotted arrows represent indirect effects, and ⊥ denotes inhibition. Regulatory interactions are marked as follows: +p for phosphorylation and -p for dephosphorylation.

**Figure 5:**
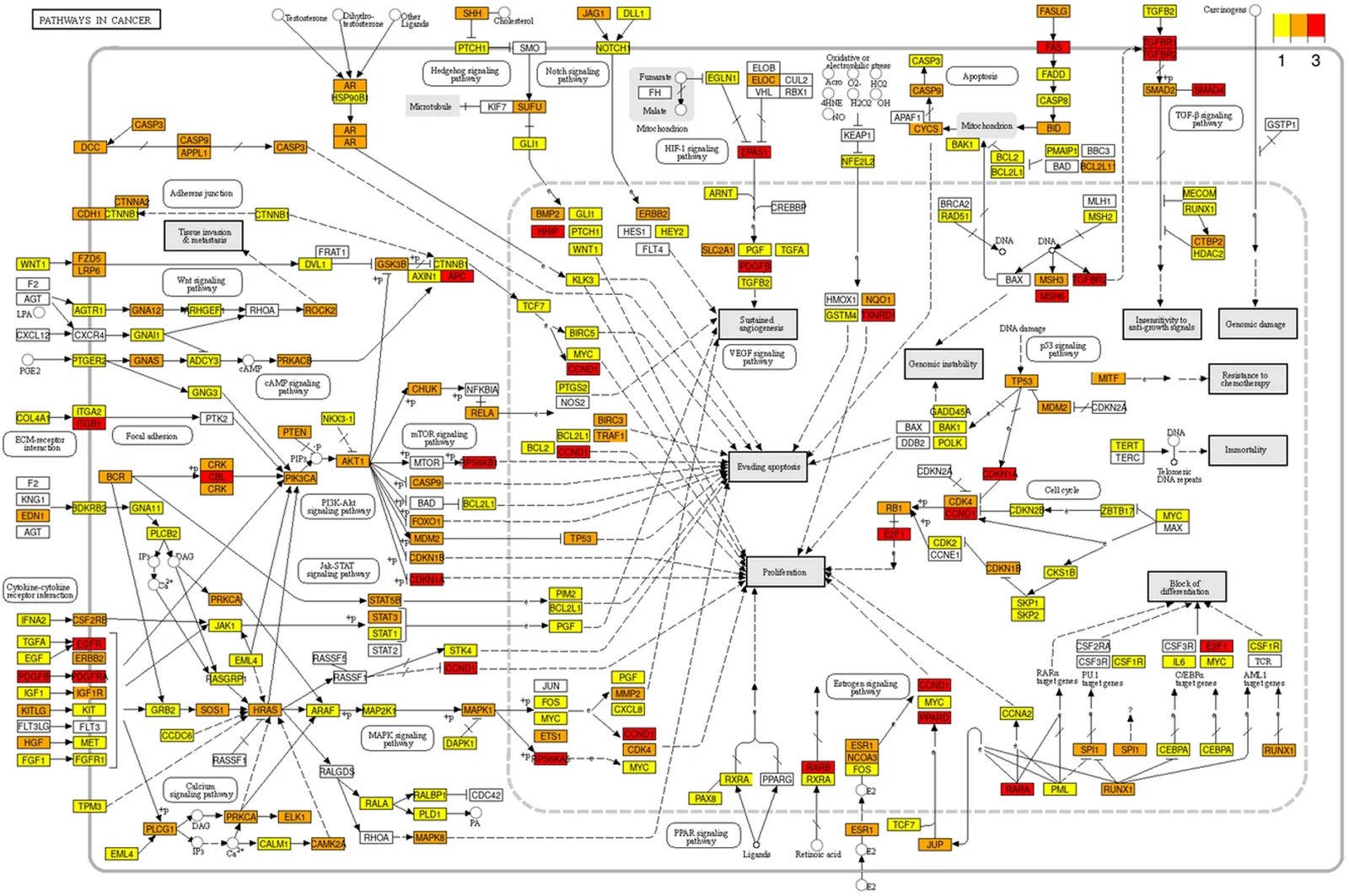
KEGG cancer-related pathways showing predicted target genes of 6 differentially expressed miRNAs. Genes identified are highlighted in color: yellow—target of one miRNA, orange—target of two miRNAs, and red—target of three miRNAs. Solid arrows, activation; dotted arrows, indirect effect; ⊥, inhibition. Letters on lines denote the type of regulation as follows: +p, phosphorylation; -p, dephosphorylation.

The regulated gene targets within the molecular processes of the cancer-related KEGG pathways are illustrated in Figs 4 and 5. These figures clearly demonstrate that most genes in these pathways are controlled by more than one DE miRNA.

GO enrichment analysis was performed to elucidate the biological functions of the DE miRNAs by examining the roles of their target genes in biological processes, cellular components, and molecular functions. The target genes were significantly enriched in 758 and 503 GO terms for the sets of 16 and 6 DE miRNAs, respectively. The top 20 statistically significant GO results from both analyses are presented in Supplementary Fig. S2, with the most enriched terms being protein binding (molecular function) and the cytoplasm and nucleus (cellular component).

## 4. Discussion

Epidemiological data have recently linked radiation exposure to increased cancer risk in patients with CHD due to IR exposure, a risk that may be further amplified by genetic susceptibility and the cumulative effect of repeated medical procedures [2]. The underlying biological mechanisms remain only partially understood, highlighting the need for further comprehensive biological studies to elucidate the response to low-dose IR and to pave the way for more effective strategies to reduce risk [9, 10]. This study presents the first global analysis of blood microRNAs in patients with CHD following radiation exposure from CC. Using next-generation sequencing, we identified 16 miRNAs that were DE immediately after the procedure and remained altered 1 year later. Additionally, six other miRNAs showed significant dysregulation 1-year post-procedure compared to both baseline and immediate post-procedure levels. To validate these findings, RT-qPCR analysis was performed on six selected miRNAs in an independent group. The results partly confirmed the sequencing findings, with four miRNAs demonstrating reliable expression patterns, while the remaining two showed consistent trends that did not reach statistical significance, likely due to low detectability.

Functional enrichment analysis was conducted to explore the potential biological roles of the dysregulated miRNAs. This analysis identified key disease pathways and target genes, with cancer-related signaling pathways, such as the MAPK pathway and others regulating the cell cycle and genomic stability, emerging as the most significantly associated. These pathways are essential for controlling growth, survival, proliferation, and metastasis [18], and their dysregulation is closely linked to cancer development and progression [18]. Overall, our findings indicate that radiation exposure from pediatric CC may result in specific dysregulation of miRNAs in the blood, which, in turn, may affect gene expression in pathways closely linked to oncogenesis.

However, causal relationships and dose-response effects have not been established. In our study, radiation exposure was assessed using commonly reported dose indicators (dose area product and total fluoroscopy time) recorded at the time of examination. It is well known that these indicators can vary widely depending on individual procedures, limiting the usefulness of a single, standard measure across all patients [8]. Therefore, more accurate evaluation of dose-response relationships may require individualized dose estimates, including organ-specific doses derived from patient-specific data.

The absence of a clear dose–response relationship in miRNA expression may also reflect biological mechanisms associated with low-dose radiation exposure, including interindividual variability in genetic and epigenetic regulation that influences miRNA biogenesis and stability, resulting in heterogeneous molecular responses to similar radiation doses. In addition, selective elimination of damaged cells and differences in lymphocyte lifespan and turnover may attenuate or obscure dose-dependent signals. Although the observed miRNA changes are significantly associated with cancer-related cellular pathways, their functional consequences and clinical relevance to radiation-induced carcinogenesis remain uncertain. Accordingly, these miRNA expression patterns should be regarded as exploratory and hypothesis-generating rather than definitive biomarkers.

### 4.1. Alterations of miRNA in cancer

MicroRNAs play central roles in carcinogenesis, functioning as oncomiRs that promote tumor growth or as tumor-suppressor miRNAs that inhibit oncogene expression, thereby influencing processes such as growth, invasion, metastasis, and angiogenesis [11]. Depending on the cancer type and cellular context, some miRNAs, including those examined in our validation group, can exert both oncogenic and tumor-suppressive effects [11].

The expression of miR-32, miR-144-3p, and miR-374a-3p is altered in many cancer types and can act either as a tumor suppressor or an oncogene, depending on the tissue and target pathway [19–21].

Conversely, let-7f-5p, miR-125a-3p, and miR-760 act primarily as tumor suppressors in various cancers, regulating proliferation, apoptosis, migration, and invasion by downregulating oncogenic targets and key signaling pathways [22–25]. Their reduced expression can enhance oncogenic activity, promoting tumor formation, progression, and aggressive traits such as increased invasion, metastasis, and poor prognosis, while restoration may have therapeutic potential [22–25]. Additionally, changes in miRNA expression may reveal molecular mechanisms of cancer development and progression following carcinogenic exposures, including IR, and serve as sensitive indicators of both acute and chronic environmental exposures [12, 26].

### 4.2. miRNA dysregulation in response to ionizing radiation exposure

Dysregulation of miRNA expression across various cell types and tissues has a pivotal role in cellular responses to IR, influencing key biological processes such as DNA damage repair, apoptosis, cell cycle regulation, and cell proliferation [11, 27].

Evidence shows that IR induces distinct changes in miRNA expression in human cells and serum [28–30]. These miRNAs can be up- or downregulated shortly after exposure, depending on cell type and context, with levels often returning to baseline over time [28–30]. While direct studies on the specific miRNAs in our validation group and low-dose radiation are limited, existing research supports their broader roles in radiation response and regulation of pathways relevant to radiotherapy [28–30].

MiR-32, miR-125a-3p, hsa-miR-144-3p, miR-374a-3p, and miR-760 have been implicated in modulating cellular responses to IR in various cancer cell lines, potentially affecting both radiosensitivity and radioresistance [31–35]. Let-7f-5p, a member of the tumor-suppressor let-7 family, shows radiation-responsive expression *in vitro* and *in vivo* [28, 29, 36]. Functionally, it regulates pathways involved in cell survival and apoptosis, including the PI3K/AKT/COX2 axis, and suppresses RAS, a key GTPase controlling growth and differentiation, thereby influencing the overall cellular response to IR [29, 30]. The let-7 family, including let-7f-5p, is consistently downregulated following IR exposure across multiple cell lines [25, 37]. Our results confirm long-term downregulation of let-7f-5p, in line with previous studies. This suppression may overactivate oncogenes and disrupt cell cycle regulation, differentiation, and apoptosis [37, 38]. Overall, let-7f-5p emerges as a key radiation-responsive tumor-suppressor miRNA, underscoring its potential as a biomarker of radiation exposure [28, 29].

However, several limitations of the present study should be acknowledged. First, due to the small cohort size and the fact that repeated measures were not explicitly modeled, the results should be interpreted with caution regarding potential false positives and the stability of identified signatures. Without integration of transcriptomic data, mechanistic interpretations of the observed miRNA patterns remain speculative. Persistent dysregulation of key miRNAs linked to cancer-related pathways does not necessarily indicate pathology, but may reflect a role in maintaining cellular homeostasis, potentially as a protective adaptation. Future studies are needed to clarify the biological relevance of these miRNA signatures, especially their downstream targets in the context of radiation response.

Second, the limited size of the validation group limits the ability to fully capture the biological and clinical heterogeneity of the study population and reduces the robustness of correlations between miRNA expression and clinical or radiation exposure parameters.

Third, the observed changes in microRNA expression cannot be attributed solely to IR, as other factors may have contributed, including anesthesia or sedation, procedural stress, and particularly the use of iodinated contrast media during cardiac catheterization. Higher tissue iodine concentrations are known to increase absorbed radiation dose via photoelectric interactions that generate secondary electrons and induce DNA damage, notably double-strand breaks [39].

In conclusion, children with CHD undergoing cardiac catheterization show changes in circulating miRNAs associated with pathways annotated as cancer-related. The longitudinal study design, incorporating baseline, immediate post-procedure, and 1-year follow-up samples, is a notable strength, enabling assessment of temporal stability. While these observations suggest potential associations with radiation exposure, their biological significance and long-term health implications remain uncertain and warrant further investigation.

## Supplementary Material

bgag032_Supplementary_Data

## Data Availability

Small RNA sequencing data of the discovery cohort are available from the corresponding author on reasonable request.
