# Supplementary material for: Exploring potential associations between low-dose cardiac catheterization radiation and blood microRNA changes in children with congenital heart disease
Source: Carcinogenesis. 2026 May 25;47(3):bgag032. doi: 10.1093/carcin/bgag032 (PMC13583624; doi:10.1093/carcin/bgag032)
Supplement: Supplementary file 1 [file bgag032_supplementary_data.zip › Supplementary File ( Legend supplementary figures).docx]

**Legend of figures**

**Figure S1.** Study design and schematic workflow for the miRNA sequencing analysis.

**Figure S2.** An illustrative example of sequence length distribution across three time points in one patient with CHD.

**Figure S3.** GO enrichment analysis of the target genes related to the differentially expressed miRNAs: a) 16 DE miRNAs at both times after CC procedures b) 6 DE miRNAs after 1 year from the procedure compared to baseline and immediately after the procedure.

**Table S1.** Summary of the data production

| **Sample** | **Reads** | **Bases** | **Error rate (%)** | **Q20 (%)** | **Q30 (%)** | **GC content (%)** |
| --- | --- | --- | --- | --- | --- | --- |
| 1 T_0_ | 12802713 | 0.64G | 0.01 | 99.18 | 97.61 | 55.43 |
| 1 T_1_ | 10892626 | 0.54G | 0.01 | 99.33 | 97.78 | 54.94 |
| 1 T_2_ | 12359601 | 0.62G | 0.01 | 99.36 | 97.97 | 55.29 |
| 2 T_0_ | 15673685 | 0.78G | 0.01 | 99.42 | 97.80 | 54.48 |
| 2 T_1_ | 17840522 | 0.89G | 0.01 | 99.36 | 97.84 | 54.92 |
| 2 T_2_ | 14656593 | 0.73G | 0.01 | 99.30 | 97.12 | 54.43 |
| 3 T_0_ | 16611984 | 0.83G | 0.01 | 99.39 | 98.02 | 55.95 |
| 3 T_1_ | 15541143 | 0.78G | 0.01 | 99.41 | 98.03 | 54.77 |
| 3 T_2_ | 16498775 | 0.82G | 0.01 | 99.12 | 96.61 | 55.05 |
| 4 T_0_ | 15830033 | 0.79G | 0.01 | 99.28 | 97.32 | 55.53 |
| 4 T_1_ | 10831155 | 0.54G | 0.01 | 99.42 | 98.07 | 55.27 |
| 4 T_2_ | 9581331 | 0.48G | 0.01 | 99.30 | 96.99 | 55.07 |
| 5 T_0_ | 12119594 | 0.61G | 0.01 | 99.14 | 97.16 | 55.74 |
| 5 T_1_ | 10557682 | 0.53G | 0.01 | 99.26 | 97.18 | 55.83 |
| 5 T_2_ | 9339623 | 0.47G | 0.01 | 99.23 | 97.03 | 56.58 |
| 6 T_0_ | 13491544 | 0.67G | 0.01 | 99.24 | 96.99 | 55.07 |
| 6 T_1_ | 9779716 | 0.49G | 0.01 | 99.24 | 97.05 | 55.22 |
| 6 T_2_ | 13002964 | 0.65G | 0.01 | 99.23 | 97.03 | 54.91 |
| 7 T_0_ | 11889903 | 0.59G | 0.01 | 99.18 | 96.76 | 54.95 |
| 7 T_1_ | 13601020 | 0.68G | 0.01 | 99.31 | 97.47 | 55.07 |
| 7 T_2_ | 14189576 | 0.71G | 0.01 | 99.31 | 97.44 | 55.46 |
| 8 T_0_ | 12033696 | 0.60G | 0.01 | 99.23 | 97.33 | 58.30 |
| 8 T_1_ | 12206844 | 0.61G | 0.01 | 99.26 | 97.30 | 58.40 |
| 8 T_2_ | 11331645 | 0.57G | 0.01 | 99.32 | 97.53 | 59.25 |
| 9 T_0_ | 8043408 | 0.40G | 0.01 | 99.17 | 96.87 | 55.60 |
| 9 T_1_ | 11423107 | 0.57G | 0.01 | 99.24 | 97.08 | 54.69 |
| 9 T_2_ | 9192871 | 0.46G | 0.01 | 99.28 | 97.30 | 56.34 |
| 10 T_0_ | 9207295 | 0.46G | 0.01 | 99.27 | 97.18 | 54.57 |
| 10 T_1_ | 10498393 | 0.52G | 0.01 | 99.39 | 97.89 | 54.86 |
| 10 T_2_ | 12340370 | 0.62G | 0.01 | 99.29 | 97.28 | 55.02 |
